# Potential of the beneficial fungus *Trichoderma* to enhance ecosystem-service provision in the biofuel grass *Miscanthus* x *giganteus* in agriculture

**DOI:** 10.1038/srep25109

**Published:** 2016-04-27

**Authors:** Ivan Chirino-Valle, Diwakar Kandula, Chris Littlejohn, Robert Hill, Mark Walker, Morgan Shields, Nicholas Cummings, Dilani Hettiarachchi, Stephen Wratten

**Affiliations:** 1 Bio-Protection Research Centre, PO Box 85084, Lincoln University, Lincoln 7647, New Zealand

## Abstract

The sterile hybrid grass *Miscanthus* x *giganteus* (*Mxg*) can produce more than 30 t dry matter/ha/year. This biomass has a range of uses, including animal bedding and a source of heating fuel. The grass provides a wide range of other ecosystem services (ES), including shelter for crops and livestock, a refuge for beneficial arthropods, reptiles and earthworms and is an ideal cellulosic feedstock for liquid biofuels such as renewable (drop-in) diesel. In this study, the effects of different strains of the beneficial fungus *Trichoderma* on above- and below-ground biomass of *Mxg* were evaluated in glasshouse and field experiments, the latter on a commercial dairy farm over two years. Other ES benefits of *Trichoderma* measured in this study included enhanced leaf chlorophyll content as well as increased digestibility of the dried material for livestock. This study shows, for the first time for a biofuel feedstock plant, how *Trichoderma* can enhance productivity of such plants and complements other recent work on the wide-ranging provision of ES by this plant species.

There is increasing interest in the role of appropriate biodiversity in agriculture, delivering multiple ecosystem services (ES)^1^. One of these is the provision of food and fibre, to which can be added biofuels. In that context, by the end of this century, anthropogenic greenhouse gas emissions are predicted to have increased global mean surface temperatures by between 1.7 °C and 4.8 °C^2^, across all scenarios. The two most significant contributors of these gases are fossil fuel combustion (57%) and land use change (17%)^3^. In the light of binding emissions reduction targets and recent international policy discussions^4^, low-carbon energy sources have been sought. Commercially viable fossil fuel replacements must be energy-dense, compatible with existing technologies and easily transportable. These demands have led to the development of liquid biofuels produced from food and energy crops as well as from waste products^5^. Mature biofuel markets have developed over the past two decades, incentivised not only by the drive to reduce carbon emissions, but also for energy security, rural development and reducing dependence on mineral oil.

Advanced biofuel feedstocks such as *Miscanthus* x *giganteus* (*Mxg*) Greef & Deuter ex Hodkinson & Renvoie, a sterile hybrid between *M. sacchariflorus* (Maxim.) Hack and *M. sinensis* Anderss., maximise biomass production by utilising C4 photosynthesis, have a prolonged canopy duration, are highly resistant to pests and diseases and undergo rapid spring growth^6^.

Such second-generation, non-food crops can be readily integrated into sustainable agricultural systems. For example, the concept of combined Food, Energy and Ecosystem Services (CFEES) utilises the ecosystem services delivered by energy crops to minimise inputs to spatially adjacent food crops^7^. In a modified CFEES agricultural system in Canterbury, New Zealand, *Mxg* generated sixteen ES^5^. This plant grows up to 2 m per annum and has the highest yield/ha of all current second-generation biofuel feedstocks^8^. The plant also has the greatest energy use efficiency of biofuel crops^9^. Grown as a shelterbelt on dairy farms, *Mxg* plots protect pasture grasses from adverse weather effects, increasing yields, organic mineralisation rates, earthworm abundance and associated biodiversity, while also permitting the continuing use of pivot irrigators in, for example, intensive dairy production. This is because the stems are resilient, bending but not breaking and promptly returning to their original form. Use of *Mxg* in this manner reduces greenhouse gas emissions from fertiliser application by enhancing fertiliser retention by pasture grasses^5^.

This study investigates the effects of the application of root endophytic *Trichoderma* isolates on the growth of a second-generation energy crop (*Mxg*). *Trichoderma* colonisation promotes growth across a broad range of species, including grasses such as wheat^10^, perennial ryegrass^11^, maize^12^, barley^10^, and sugarcane^13^. Growth promotion is most significant under suboptimal conditions where *Trichoderma* association is correlated with enhanced nutrient uptake and mineral solubilisation^14^. *Trichoderma* colonisation is also associated with increased root biomass and depth^15^, which may assist soil organic carbon retention.

The aim of the current work was to evaluate a mixture of *Trichoderma* isolates for enhancement of key ES in *Mxg* in glasshouse pot trials (with commercial potting compost or field-collected soil). Research was then extended to a two-year trial site on a commercial dairy farm where *Mxg* growth rates were compared between three *Trichoderma* isolate mixtures and a control.

## Materials and Methods

### Glasshouse experiment 1. Effects of a mixture of *T. atroviride* isolates on the growth of *Miscanthus* x *giganteus* in field-collected soil

#### Experimental design

A glasshouse study was conducted to investigate the effects of *T. atroviride* isolate mixture PR7 on the growth of *Mxg* in field-collected soil containing the pathogenic fungus *Rhizoctonia solani* Kühn^11^. The presence of *R. solani* in the collected soil was confirmed at the start of the experiment using a bioassay involving pre-germinated seeds of radish (*Raphanus sativus* cv. Rex)^16^.

The soil was collected from a Lincoln University field (43° 38′ 46” S; 172° 27′ 10” E), passed through a 3 mm sieve and mixed with pumice (3:1 soil/pumice). Four *T*. *atroviride* isolates (FCC 01, FCC 02, FCC 04, FCC 05), comprising mixture PR7, from the BCMCC culture collection at Lincoln University, New Zealand, were grown together in a sterile mixture of wheat bran and peat (3:1 wheat bran/peat). One gram of each mixture (2 × 10^8^ CFU/g) was added to 800 g of soil and thoroughly mixed. *Mxg* rhizomes c. 3 cm in height and width were transplanted into 1 L pots containing the inoculated soil/pumice mixture and arranged in a randomised block design comprising five replicates each of the *Trichoderma* isolate mixture and controls (10 pots in total). The pots were kept in a glasshouse without artificial light and with variable temperature (mimicking to some extent field conditions) from December 2013 to April 2014. The watering regime was the same for all pots and optimum soil water levels were maintained. Water was added until it began to drain from the bottom of the pots. Five months after establishment, the soil in the rhizosphere in the pots was sampled to quantify *Trichoderma* colonisation using soil-dilution plating on a *Trichoderma* selective medium (TSM)^17^. Plants were then transferred to larger pots (8.5 L), each filled with 5 kg of fresh soil/pumice mixture and allowed to grow for a further 12 months under natural day length (May 2014 to April 2015).

#### Plant growth assessment and analyses

After the 17-month growing period, numbers of shoots (main stem and tillers) were counted for each pot and then cut at soil level and placed in paper bags. Rhizosphere soil was sampled for a final assessment of *Trichoderma* colonies. To do this, roots were carefully removed from the rhizome and washed. 100 fine root pieces (2–3 mm) from each pot were surface-sterilised and plated on a TSM to obtain percentage *Trichoderma* root colonisation. The remaining root tissues were separated from rhizomes and bagged. These, as well as the rhizomes themselves and shoots, were separately dried at 60 °C for 48 h for each plant and dry weights were recorded.

### Glasshouse experiment 2. Effects of a range of *Trichoderma* mixtures on *Miscanthus* x *giganteus* in standard potting mix

#### Experimental design

Seventeen *Trichoderma* isolates including isolates of *T. atrobrunneum* F.B. Rocha, P. Chaverri & W. Jaklitsch, *T. atroviride* P. Karst.*, T. crissum* Bissett*, T. harzianum* Rifai, and *T. koningiopsis* Samuels, C. Suárez & H.C. Evans from the BCMCC culture collection at Lincoln University were grown on malt-yeast extract agar (1% malt extract, 0.1% yeast extract, 2% agar) plates to produce conidial inocula for a pot experiment with *Mxg* rhizomes. Five treatments (mixtures) containing equal proportions of different isolates were prepared in an aqueous suspension of 0.01% Tween 80 to give a total of 10^6^ conidia per ml. One mixture (PR7) was the same as that used in Experiment 1 (see Tables 1 and 2).

In May 2014, a randomised block greenhouse pot trial was established comprising twenty replicates for each of the five treatments (mixtures) and of an untreated control. *Mxg* rhizomes were soaked overnight in the aqueous suspension of each of the five *Trichoderma* isolate mixtures and then planted in 2.5 L pots containing 80% composted bark, 20% pumice (grade 1–7 mm), Osmocote fertiliser, horticultural lime, and Hydraflo (a wetting agent). A 16/8 h day-length regime was maintained in the glasshouse.

#### Plant growth assessment and analyses

Monthly assessments of shoot number and height (mean of the five tallest shoots) were carried out on each plant between July and September 2014. The dry weight of shoots and of the rhizomes with roots attached was recorded in early October after oven drying the material at 65 °C for 2 days.

Near infra-red spectroscopy (NIR; FOSS 6500NIR System) was conducted on dry and finely ground samples of the shoots and roots to analyse potential nutritional values for grazing livestock. Roots were cut from each rhizome and for each treatment, all roots were combined. The same procedure was carried out for all the shoots in each replicate. This was because the biomass/replicate was not enough for analysis of individual plants. The parameters were water-soluble carbohydrate, protein %, organic matter, organic matter digestibility, dry organic matter digestibility, dry matter digestibility, acid detergent fibre, and neutral detergent fibre.

In September 2014 (four months after inoculation), a Minolta SPAD-502 chlorophyll meter was used to measure the green colour of the plants under each treatment. Three readings (on a scale of 0 to 100) were obtained and averaged from the five tallest leaves of each plant. The chlorophyll data were expressed as percentage differences compared with control. This chlorophyll quantification method correlates very strongly with saturation chroma C* and hue angle H°^18^.

### Field experiment. Assessment of *Miscanthus* x *giganteus* growth with *Trichoderma* isolate mixtures

#### Experimental design

A randomised block experiment with five blocks and four treatments was established in November 2014 along the edge of one field on a commercial dairy farm (43° 32′ 15” S; 172° 16′ 16” E). The soil comprised Chertsey stony silt loam and Lismore silt loam. The *Trichoderma* isolate mixtures PR5, PR6 and PR7 used were selected based on the results obtained from Glasshouse Experiments 1 and 2. Each of the twenty replicates (7 m × 25 m) received 175 *Mxg* plants. Experimental plants in each replicate were previously treated with one of the above three mixtures (as explained in Glasshouse Experiment 2), controls were untreated.

#### Plant growth assessment

The number of shoots and height of the tallest shoot/plant were calculated.

#### Statistical analyses

Randomised block analyses of variance were carried out on data from each of the three experiments (Glasshouse 1 and 2 and Field).

## Results

### Glasshouse experiment 1. Effects of a mixture of *T. atroviride* isolates on the growth of *Miscanthus* x *giganteus* in field-collected soil

There were no significant differences between PR7 and the untreated control for the number of colony forming units (CFUs) after 5 months (Table 1). However, that parameter and the percentage of roots colonised were significantly higher under PR7 compared to the control after 17 months.

Similarly, shoot, root and rhizome dry weights were significantly higher (p < 0.05) in the PR7 treatment after 17 months’ growth, while the number of shoots and plant height did not differ significantly from the control at that stage (Table 3). This can be explained by the fact that plant growth of *Mxg* in the pots was slow during the first 4–5 months when growth of only fine roots was observed. Differences between treatments became apparent between 6 and 12 months and this can be attributed to increased rhizome growth in some treatments.

### Glasshouse experiment 2. Effects of a range of *Trichoderma* isolate mixtures on *Miscanthus* x *giganteus* in standard potting mix

#### Plant height

**Figure 1:**
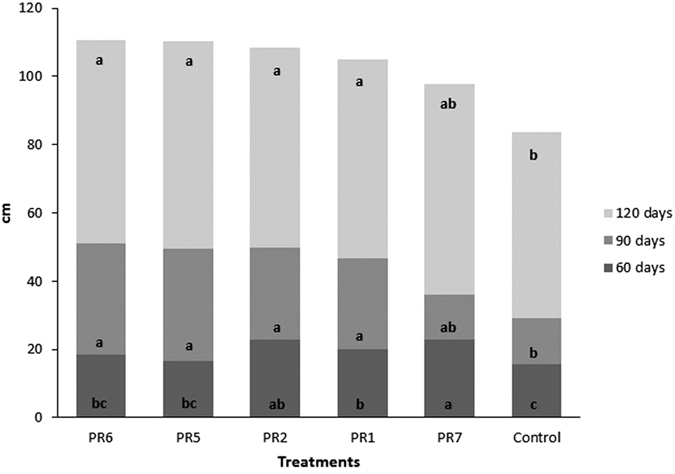
Glasshouse experiment 2. Mean height (cm) of *Miscanthus* x *giganteus* in the five *Trichoderma* isolate mixtures and in the control, assessed 60, 90 and 120 days after planting. Different letters within each assessment date indicate significant plant-height differences (p < 0.05).

**Figure 2:**
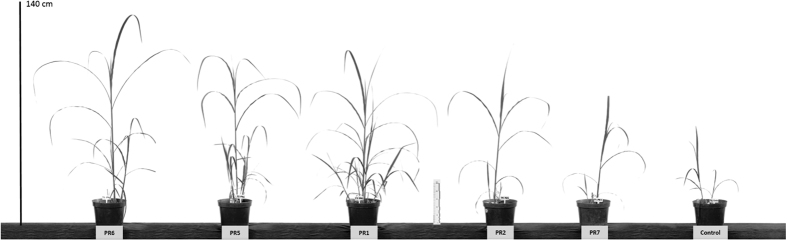
Glasshouse experiment 2. An illustration of the heights of *Miscanthus* x *giganteus* plants 90 days after planting in the greenhouse. One plant was randomly selected from each treatment, which were, from left to right: PR6, PR5, PR1, PR2, PR7 and the control.

The untreated control plants were consistently smaller than those in the other treatments at 60, 90 and 120 days after planting (Fig. 1). Plants with the PR7 mixture were significantly higher (p < 0.05) at 60 days after planting than the other treatments (except for PR2). Plants in the PR6 mixture were taller than the control at 90 and 120 days after planting (Fig. 2).

#### Shoots

There were no significant differences in shoot number among the treatments at 60 days after planting. At 90 days, PR1 plants had significantly more shoots than the control, while at 120 days, PR5, PR1 and PR6 had more shoots than PR7 and the control.

#### Leaf green colour

The leaf green colour readings obtained from *Mxg* in September in the PR6 treatment were 11% higher than in the control, which had the lowest values. Other treatments increased mean percentage chlorophyll content compared with the control by 3% to 8% (PR1 = 3%, PR5 = 5%, PR2 = 8%, PR7 = 8%, and PR6 = 11%).

#### Dry weight

**Figure 3:**
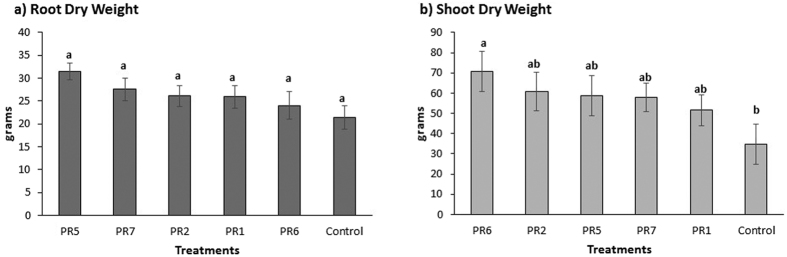
Glasshouse experiment 2. Dry weight (g) of *Miscanthus* x *giganteus* for (**a**) roots and (**b**) shoots assesed 5 months after planting. The error bars represent standard errors of means. Different letters indicate that means differ significantly (p < 0.05).

*Trichoderma* treatment had no effect on root dry weight, although control weights were the lowest (Fig. 3a). However, for shoot dry weight, PR6 plants had the highest dry weight of all treatments and were significantly higher than the control (Fig. 3b).

#### Plant nutritional quality

There were no significant differences between treatments for any parameters in roots and shoots, except for water-soluble carbohydrate in shoots, where the PR1 treatment was significantly (p < 0.05) higher than the control (8.7% and 6.8%, respectively).

### Field experiment. Assessment of *Miscanthus* x *giganteus* growth with *Trichoderma* isolate mixtures

At 5 months after planting, plant height was greatest in the PR5 treatment and this differed significantly from the control and PR6 treatments (p < 0.05). For shoot number, there was no significant effect of any treatment (p > 0.05) (Table 4).

## Discussion

Greenhouse gas emissions released mainly from first-generation biofuel production can result from initial land clearance prior to planting, high application of inputs such as herbicides, fungicides, pesticides and fertilisers, and of the energy required to harvest and process the fuels. Biofuel yield divided by these emissions is defined as global warming payback time, or GWPBT. In light of food security and GWPBT, the sustainability potential of some biofuels is considerably reduced. However, the giant grass *Mxg* is capable of producing a higher yield with lower inputs and so has a lower GWPBT than many other biofuel feedstocks (Table 5).

Here, we have demonstrated that application of *Trichoderma* species and isolates can increase growth in a second generation biofuel feedstock. Specifically, increases in chlorophyll concentration, shoot dry weight and plant height were observed in *Mxg* plants treated with *Trichoderma* isolate mixture PR6. These results are consistent with *Trichoderma*-induced growth promotion in many other plant systems. For example, *T. atroviride* can increase both root and shoot growth in tomato^19^ and oilseed rape^20^. This latter result was from treatment with *T. atroviride* isolate FCC 01, which was present in the PR7 mixture here. In our study, this mixture gave statistically significant increases in height, but not in biomass in standard potting mix. This treatment did, however, give increased root, shoot and rhizome biomass in soils previously infected with *Rhizoctonia* compared with untreated plants. Similarly, *T. harzianum* induces growth promotion in a number of commercially grown species^21^; however, no *T. harzianum* isolates used in this study had been evaluated in this way previously.

In the field, *Trichoderma* isolate mixtures PR5 and PR6 significantly led to greater plant height. Destructive shoot and root biomass sampling has yet to be conducted, although for field-soil pot trials, height and biomass measurements were correlated. Increased plant height suggests enhanced vigour. This is crucial for establishment of *Mxg* crops on marginal land, utilization of which will be an important factor in future biofuel production^22^. However, Littlejohn *et al.*^5^ showed, using a resource economics analysis, that the value of ES delivered by *Mxg* can negate potential disadvantages of using agricultural land. Continuing evaluation of field trials will allow for further investigation of the effects of *Trichoderma* treatment on abiotic and biotic stressors, the latter including plant diseases.

Colonisation of plant roots by *Trichoderma* species has been shown to promote plant growth through a range of different mechanisms^21^. In some cases, increased growth may result from *Trichoderma* strains limiting the effects of plant pathogens by direct mycoparasitism^23^, production of antimicrobial compounds^24^, competition for nutrients and space in the rhizosphere^25^,^26^, and induced resistance in the host plant^27^. *Trichoderma* species have also been reported to produce diverse secondary metabolites that promote plant growth, including indole acetic acid (IAA) and auxin analogues^28^, or other growth-regulating compounds such as 6-pentyl-alpha-pyrone (6PP) and harzianolide^29^. Plant growth may also be enhanced by the solubilisation of mineral nutrients, for example *T. harzianum* strains were shown to increase the availability of plant nutrients by solubilising organic and inorganic phosphates, Fe_2_O_3_, CuO, metallic Zn, and MnO_2_^30^,^31^. Different *Trichoderma* isolates may elicit plant growth promotion through one or more of these potential mechanisms in a strain-specific manner, i.e. the ability to use a given mechanism is not necessarily a characteristic of particular *Trichoderma* species^21^,^28^.

The mixtures of *Trichoderma* isolates used in our experiments were previously selected on the basis of their strong performance in promoting growth and/or supressing fungal disease in a range of other plants^21^. Use of isolate mixtures allows incorporation of isolates with different benefits to the host plant (e.g. mixture PR6 includes growth-promoting and disease-suppressing isolates). Another advantage of using *Trichoderma* isolate mixtures is that individual isolates may show varying activity in different plants, so a combination of isolates in a mixture allows the development of plant protection agents that may be used successfully in a range of crops. Within our work, it is difficult to speculate on which specific isolates are responsible for the growth promotion effects observed. Further work should include identification of whether these effects result from any synergistic interactions between different isolates in a mixture, or are predominantly due to the action of individual isolates.

Fungal pathogens and arthropod pests cause economically significant damage to grass crops worldwide^32^. Concerns have been raised that graminaceous biofuel crops may be vulnerable to existing fungal diseases as well as to emerging pathogens^33^,^34^. This is an acute threat for *Mxg*, given the limited genetic diversity within the existing commercialised hybrid^35^. Treatment with the *Trichoderma* isolate mixture PR7 resulted in statistically significant increases in biomass and shoot height in soils infected with the soil pathogen *Rhizoctonia solani*. Growth promotion has also been observed following treatment with PR7 in non-infected soil in pot trials in the current study. However, it is unclear if growth promotion has mitigated the stunting effect of *Rhizoctonia* infection, or if the *Trichoderma* treatment has reduced the incidence of the *Rhizoctonia* in these trials. Many *Trichoderma* species are aggressive mycoparasites of other fungi; notably *T. hamatum* is reported to attack *Rhizoctonia*^36^ and has been used previously for biological control of fungal diseases^37^.

The biofuel potential of a range of non-food cellulosic feedstock plants is well known^5^,^6^. However, a crucial factor influencing the commercial development of such crops is energy yield/ha, their environmental impact and the economic return on investment. Future use of effective *Trichoderma* isolate mixtures as biological plant protection agents may assist in lowering inputs and increasing yields, the two components of sustainable intensification^38^.

## Additional Information

**How to cite this article**: Chirino-Valle, I. *et al.* Potential of the beneficial fungus *Trichoderma* to enhance ecosystem-service provision in the biofuel grass *Miscanthus* x *giganteus* in agriculture. *Sci. Rep.* **6**, 25109; doi: 10.1038/srep25109 (2016).

## Figures and Tables

**Table 1:** Glasshouse experiment 1 (field collected soil).

	Treatments		LSD
	PR7	Control	
CFUs (Log10) at 5 months	5.7 a	2.1 b	1.0
CFUs (Log10) at 17 months	4.4 a	1.4 b	0.9
PRC at 17 months	33.6 b	1.6 b	7.1

Mean number of colony forming units (CFUs) of *Trichoderma atroviride* and the percentage of roots colonised (PRC) in PR7 and control treatments 5 and 17 months after trial establishment. Different letters for each parameter indicate that means differ significantly between treatments (p < 0.05).

**Table 2:** Glasshouse experiment 2 (standard potting mix). Mixtures and sources of *Trichoderma* isolates used in this study.

Mixture	Isolates	Species	Plant source*	Location (NZ)
PR1	FCC 318	*T. atrobrunneum*	*Allium sativum*	Waikato
	FCC 319	*T. atrobrunneum*	n.d.	Waikato
	FCC 320	*T. atrobrunneum*	*Agapanthus* sp.	Waikato
	FCC 322	*T. koningiopsis*	*Agapanthus* sp.	Waikato
	FCC 340	*T. harzianum*	*Echium vulgare*	Mid Canterbury
PR2	FCC 49	*T.* sp.	*Cyathea dealbata*	Mid Canterbury
	FCC 55	*T. harzianum*	Liliaceae	Mid Canterbury
	FCC 362	*T. crassum*	*Hoheria sexstylosa*	Bay of Plenty
	FCC 368	*T. atrobrunneum*	*Cassia sp.*	Waikato
PR5	FCC 161	*T. harzianum*	Poaceae	Bay of Plenty
	FCC 180	*T. crassum*	*Pinus radiata*	Bay of Plenty
	FCC 275	*T. atroviride*	*Pittosporum sp.*	Bay of Plenty
	FCC 327	*T. harzianum*	*Mentha* sp.	Waikato
PR6	FCC 55	*T. harzianum*	Liliaceae	Mid Canterbury
	FCC 318	*T. atrobrunneum*	*Allium sativum*	Waikato
	FCC 327	*T. harzianum*	*Mentha* sp.	Waikato
	FCC 340	*T. harzianum*	*Echium vulgare*	Mid Canterbury
PR7	FCC 01	*T. atroviride*	Rhizosphere soil	Auckland
	FCC 02	*T. atroviride*	Rhizosphere soil	Auckland
	FCC 04	*T. atroviride*	*Ciborinia camelliae*	Wellington
	FCC 05	*T. atroviride*	Rhizosphere soil	Westland

^*^All samples were obtained from roots apart from rhizosphere soil where indicated.

**Table 3:** Glasshouse experiment 1 (field collected soil).

	Treatments	
	PR7	Control
Shoot number	5.6 a	2.8 a
Shoot dry weight (g/plant)	90.9 a	53.3 b
Root dry weight (g/plant)	77.9 a	28.8 b
Rhizome dry weight (g/plant)	58.3 a	37.5 b
Plant height (cm)	108.4 a	109.8 a

Mean shoot number/plant, shoot, root and rhizome dry weights and plant height in PR7 and control treatments. Different letters in a row indicate that means differ significantly between treatments (p < 0.05).

**Table 4:** Field experiment.

	Treatments			
	Control	PR5	PR6	PR7
Height	69 b	81.5 a	75 b	77 ab
Shoots	4.5 a	4.7 a	4.3 a	3.5 a

Mean values of height (cm) and shoot number of *Miscanthus* x *giganteus* between PR7 and control after 5 months of trial establishment. Different letters indicate that means differ significantly between treatments (p < 0.05).

**Table 5:** A comparison of candidate biofuel feedstock plant species across several parameters.

Crop	Harvestable biomass (tons/ha/yr)	Ethanol (L/ha)	Ha needed for 132.5 billion L ethanol (millions)	% of 2006 harvested US cropland: 165 million Ha (2007)	Energy output/ input ratio
Maize grain	3.1–6.4^a^	4266	31	19^b^	–
Maize stover	3.1–6.4^a^	2805	47	29^b^	–
Maize total	6.2–12.8^c^	3500–5400^d^	25–38^d^	15–23^b^	10.7^c^
Switchgrass	5.2–11.1^e^	2000–2900^d^	46–66	28–40^b^	–
*Miscanthus*	20.9–34.6^e^	8600^d^	15	9^b^	–
Prairie	3.8	1439	92.1	56^b^	–
Willow	6.2–12.8^c^	–	–	–	24.0^c^
Wheat total	7–14.4^c^	–	–	–	11.3^c^

^a^Graham, *et al.*^39^;

^b^Nickerson, *et al.*^40^;

^c^Börjesson and Tufvesson^41^;

^d^Zhuang, *et al.*^42^;

^e^Fargione, *et al.*^43^; – indicates no data.
